# Standardized digital solution with surgical procedure manager (SPM®)—an opportunity for maximizing patient safety and efficiency in ileostomy reversal?

**DOI:** 10.3389/fsurg.2023.1141017

**Published:** 2023-06-20

**Authors:** Rahel M. Strobel, Christian H. W. Schineis, Leyre Lasierra Viguri, Andrea Stroux, Sophie M. Eschlböck, Leonard A. Lobbes, Ioannis Pozios, Claudia Seifarth, Benjamin Weixler, Carsten Kamphues, Katharina Beyer, Johannes C. Lauscher

**Affiliations:** ^1^ Department of General and Visceral Surgery, Charité – Universitätsmedizin Berlin, Corporate Member of Freie Universität Berlin and Humboldt-Universität zu Berlin, Campus Benjamin Franklin, Berlin, Germany; ^2^ Department of General Surgery, Sana Klinikum Lichtenberg, Berlin, Germany; ^3^ Institute of Biometry and Clinical Epidemiology, Charité – Universitätsmedizin Berlin, Corporate Member of Freie Universität Berlin, Humboldt-Universität zu Berlin and Berlin Institute of Health, Berlin, Germany; ^4^ Department of General and Visceral Surgery, Parkklinik Weißensee, Berlin, Germany

**Keywords:** digital surgery, surgical procedure manager, digital checklist, standardization, ileostomy reversal

## Abstract

**Background:**

Standardization and digitalization are getting more and more essential in surgery. Surgical procedure manager (SPM®) is a freestanding computer serving as a digital supporter in the operating room. SPM® navigates step-by-step through surgery by providing a checklist for each individual step.

**Methods:**

This was a single center, retrospective study at the Department for General and Visceral Surgery at Charité—Universitätsmedizin Berlin, Campus Benjamin Franklin. Patients who underwent ileostomy reversal without SPM® in the period of January 2017 until December 2017 were compared to patients who were operated with SPM® in the period of June 2018 until July 2020. Explorative analysis and multiple logistic regression were performed.

**Results:**

Overall, 214 patients underwent ileostomy reversal: 95 patients without SPM® vs. 119 patients with SPM®. Ileostomy reversal was performed by head of department/attendings in 34.1%, by fellows in 28.5% and by residents in 37.4%; *p* = 0.91. Postoperative intraabdominal abscess emerged more often in patients without SPM®: ten (10.5%) patients vs. four (3.4%) patients; *p* = 0.035. Multiple logistic regression showed a risk reduction for intraabdominal abscess {Odds ratio (OR) 0.19 [95% confidence interval (CI) 0.05–0.71]; *p* = 0.014} and for bowel perforation [OR 0.09 (95% CI 0.01–0.93); *p* = 0.043] in the group with use of SPM® in ileostomy reversal.

**Conclusions:**

SPM® may reduce postoperative complications in ileostomy reversal such as intraabdominal abscess and bowel perforation. SPM® may contribute to patient safety.

## Introduction

The main objectives of surgery are high surgical quality and patient safety. Achieving the best surgical outcome with minimal postoperative complications has to be balanced against a minimal utilization of time and resources. Standardization of intraoperative processes may be the key to merge high surgical quality with no significant differences in terms of lead-operating surgeon, date of procedure and postoperative outcome.

One way to perform standardization was the introduction of the patient-safety checklist of the World Health Organization (WHO) in 2008. It consists of 20 items focussing on teamwork, communication and anticipation of adverse events (1). Another way to achieve standardization is increasing digitalization. Surprisingly, the use of computer-aided technologies in visceral surgery is rare. The surgical procedure manager (SPM®) is a digital tool designed by Surgical Process Institute (SPI) Germany GmbH in 2014 (Johnson & Johnson Medical GmbH, Norderstedt, Germany). The surgical procedure is divided in substeps which refer to the landmarks of the operation. These master processes include the following steps during surgery: patient's position on the operating table, position of surgeons, required instruments, team time out, granular steps of the surgical procedure and ordering of the next patient.

SPM® was implemented at the Department of General and Visceral Surgery at Charité—Universitätsmedizin Berlin, Campus Benjamin Franklin in 2018 in a pilot study. It has been used for ileostomy reversal, laparoscopic cholecystectomy and laparoscopic ileocecal resection. In this retrospective trial, we chose ileostomy reversal for SPM® because it is very well standardized and it is regularly performed by both residents and fellows under supervision of a senior surgeon. We hypothesized that the use of SPM® in ileostomy reversal reduces postoperative wound infections and reduces duration of surgery compared to ileostomy reversal without SPM® in an exploratory analysis.

## Material and methods

This was a single center, retrospective study at the Department of General and Visceral Surgery at Charité—Universitätsmedizin Berlin Campus Benjamin Franklin. Patients 18 years or older who underwent elective ileostomy reversal without SPM® between January 2017 and December 2017 were compared to patients who were completely operated with SPM® between June 2018 and July 2020.

**Figure 1:**
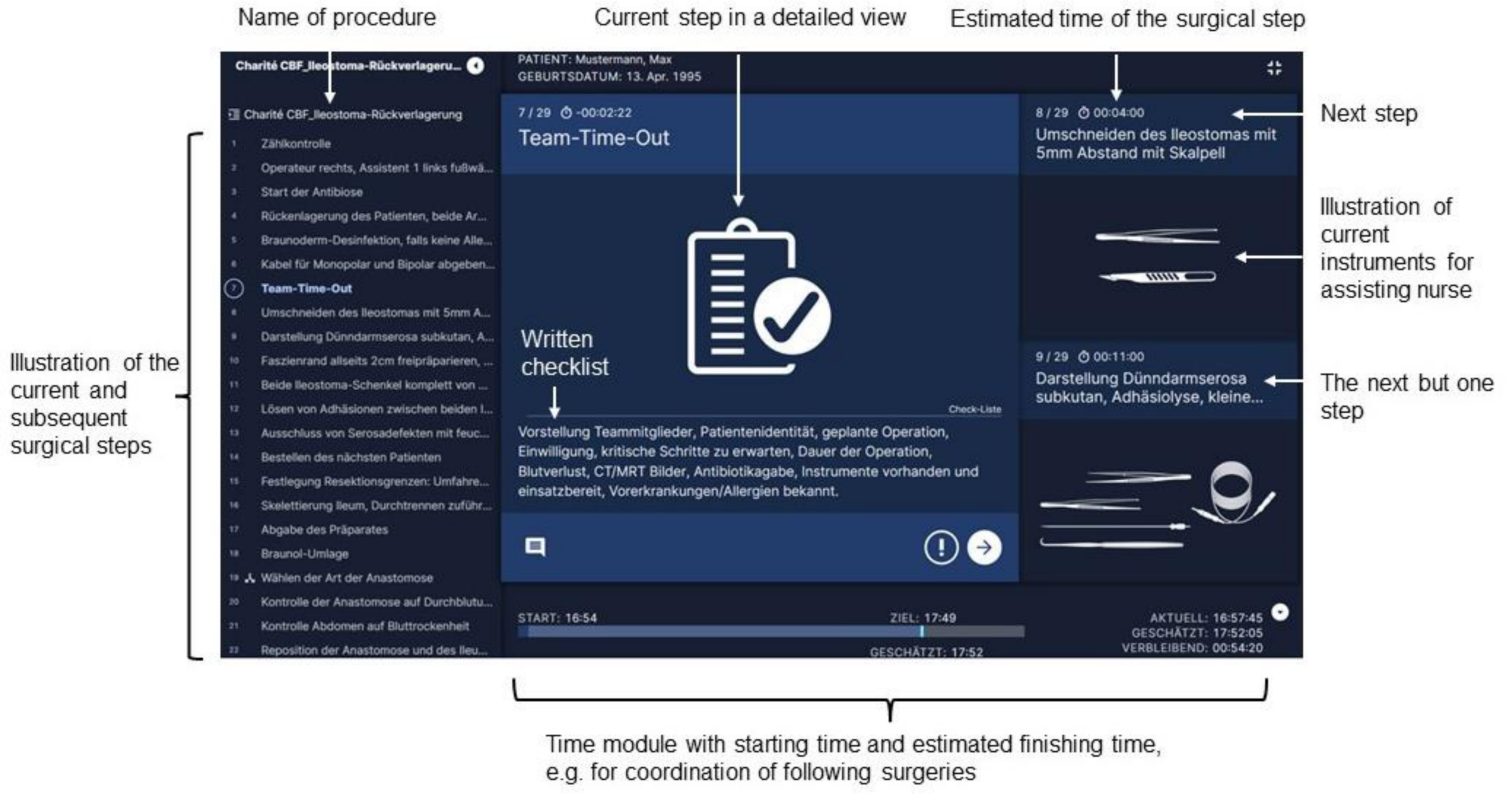
Explanation of display for ileostomy reversal of SPM® by Surgical Process Institute (SPI) Germany GmbH, Johnson & Johnson medical GmbH.

SPM® was implemented at Charité—Universitätsmedizin Berlin, Campus Benjamin Franklin in 2018 in a pilot study. It is a freestanding computer serving as a digital supporting tool in the operating room. SPM® can be controlled via foot pedal or voice control. The details of the current step appear both in written and pictorial form on the monitor. The headings of the next two steps already show up on the monitor on the right-handed side (Figure 1). Furthermore, SPM® supplies a time module at the bottom of the screen with starting time of the procedure and estimated finishing time. The estimated finishing time is being calculated from the actual time spent and the estimated time of the steps ahead. This can guide the surgeons, assisting nurses and anaesthesiologists through the operation.

We created our own digital surgical workflow (master process) for ileostomy reversal according to the standard operating procedure of our department. Bowel preparation was not administered in ileostomy reversal. A single dose of 1.5 g cefuroxime (M.P.I. Pharmaceutica, Hamburg, Germany) and 500 mg metronidazole intravenously (Braun, Bethlehem, USA) was given 30 min before skin incision in all patients. Skin preparation was performed with Braunoderm® (50% 2-propanol and 1% povidone-iodine) (Braun, Melsungen, Germany) or in case of allergy to povidone iodine with 70% 2-propanol (Braun, Melsungen, Germany).

**Figure 2:**
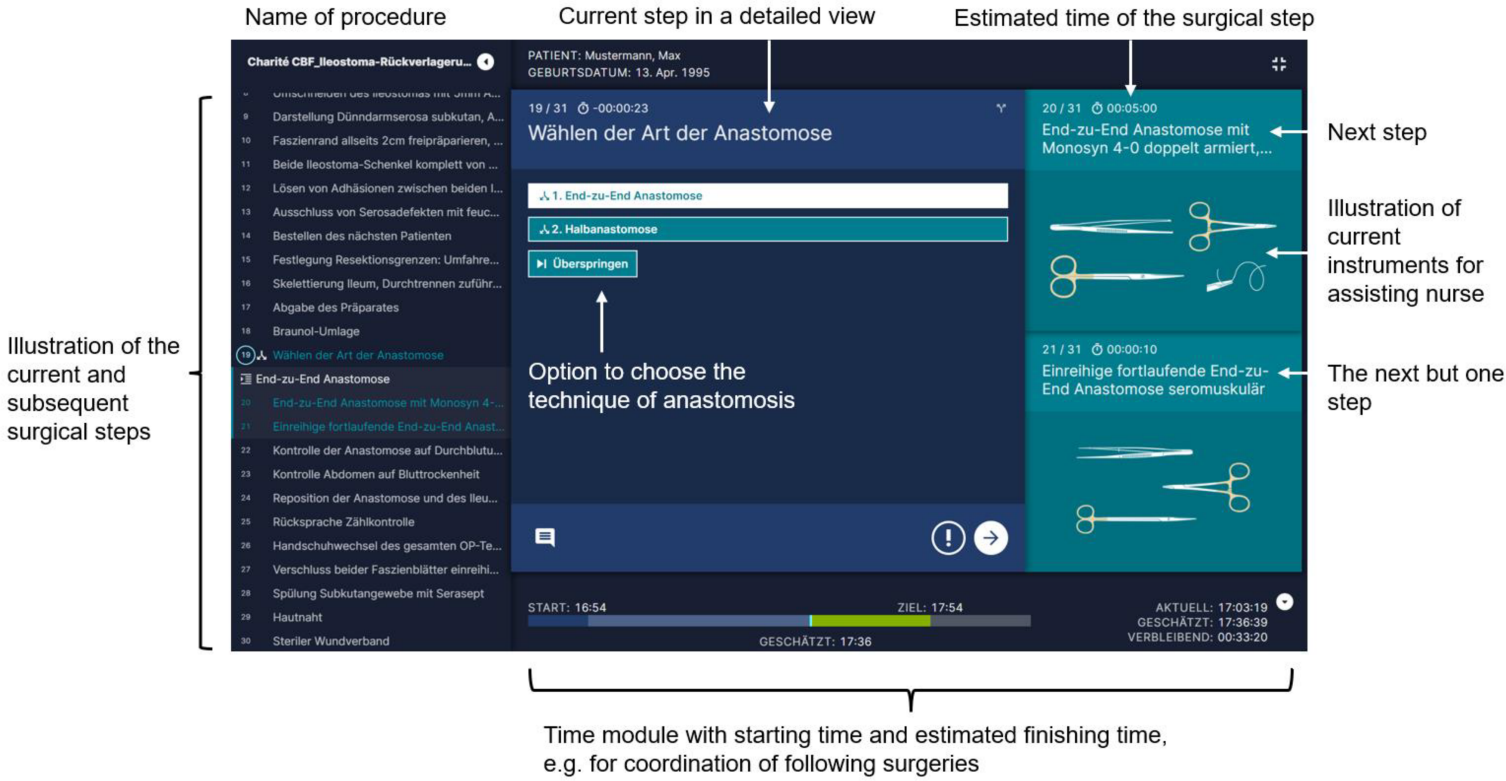
Explanation of display with the step to choose two pathways regarding the technique for anastomosis in ileostomy reversal of SPM® by Surgical Process Institute (SPI) Germany GmbH, Johnson & Johnson medical GmbH.

This study is the first to examine the use of SPM® in visceral surgery. This surgical procedure consists of 32 steps altogether. They are divided in seven phases (Table 1). The skin is incised circularly around the ileostomy to create a parastomal access. Afterwards, lysis of adhesions of small bowel and fascia is performed with a sharp scissor. Lysis of adhesions is carried on with the afferent and efferent small bowel loop. The afferent and efferent small bowel loop will then be checked for damage by putting a swab inside of the bowel loops. The ends of both small bowel loops are resected over a length of 2 cm each. The resected small bowel will be sent to the pathology for histological examination. Abdominal cloths are placed of the incision. Regarding the anastomosis in ileostomy reversal two pathways can be followed. It can be chosen between either an end-to-end-anastomosis with resection of a short segment of small bowel (preferred) or an anastomosis of the anterior wall without small bowel resection (Figure 2). Regardless of the chosen pathway, all anastomoses were performed in a hand-sewn manner. The surgeon checks the anastomosis for closeness and tightness with the fingers. Afterwards the surgical gloves of the whole operation team are changed. Abdominal drains are never placed in our department in ileostomy reversal. The fascia of the abdominal wall is closed by using slowly absorbable sutures. The subcutaneous tissue is irrigated with 0.04% polyhexanide solution considering the application time of the solution. Closure of the skin is performed by using non absorbable sutures in a linear manner.

**Table 1:** Definition of phases during ileostomy reversal.

Phase	Step	Definition of phase
1	1–7	Preoperative phase until team time-out
2	8	Skin incision, parastomal access
3	9–12	Adhesiolysis of small bowel and fascia, adhesiolysis of afferent and efferent small bowel loop
4	13–23	Resection of small bowel; selection, creation and check of anastomosis
5	24–28	Closure of abdominal wall, subcutaneous irrigation
6	29	Closure of skin
7	30–32	Dressing, pathological report

The study protocol was approved by the State Office for Health and Social Affairs Berlin (EA4/106/20). The trial was conducted in accordance with the ethical principles of the Declaration of Helsinki and the principles of Good Clinical Practice (ICH-GCP E6) (2).

Data was extracted retrospectively. Patient characteristics included underlying disease, prior operation that led to the formation of the loop ileostomy, potential synchronous treatment with immunosuppressants, comorbidities, intra- and postoperative course and postoperative complications. The primary endpoint of this study was the rate of surgical site infections (SSI) expressed as absolute number of patients and percentage. This was defined as the incidence of any SSI within 30 days after surgery according to the criteria standardized by the Center for Disease Control (CDC). Superficial incisional SSI (grade I) was defined as an infection that involves only skin and subcutaneous tissue of the incision. Deep incisional SSI (grade II) involves deep soft tissue of the incision (e.g., fascial and muscle layers) and organ/space SSI (grade III) is defined as an infection comprising any part of the body deeper than fascial/muscle layers that was opened or manipulated during an operation (3).

Secondary endpoints were the rate of re-operation, anastomotic leakage, postoperative intraabdominal abscess and postoperative bowel perforation (not involving the anastomosis) expressed as absolute number and percentage as well as the duration of procedure in minutes and length of hospital stay in days. When anastomotic leakage or intraabdominal abscess after ileostomy reversal was suspected, the patients underwent computer tomography of the abdomen. Intraabdominal abscess could then be diagnosed in the scan of the abdomen. In case of free abdominal air or radiologically assumed anastomotic leakage, the surgical team decided if surgical revision was necessary. Anastomotic leakage was then confirmed intraoperatively by inspecting the anastomosis.

Furthermore, the duration of the procedure between different levels of training (head of department/attending vs. fellow vs. resident/intern) was evaluated secondarily.

The company providing SPM® (Johnson & Johnson Medical GmbH, Norderstedt, Germany). did not sponsor the study. Johnson & Johnson was neither involved in conduct of the study, nor in acquisition and analysis of data nor in writing the manuscript.

The calculation of effect size was carried out with the help of nQuery and nTerim 4.0. An effect size of 0.423 on a two-sided significance level of 5% with 80% power in *t*-test could be detected with a sample size of 77 patients who had ileostomy reversal with SPM® vs. 105 patients without SPM®. Adjustment regarding potential confounders was achieved by using multivariate analysis.

For quantitative outcomes, statistical group comparisons were performed using the *t*-test or, in case of normality assumption being violated, Mann-Whitney *U* test for independent samples. Additional parameters were depicted according to their scale and distribution with absolute and relative frequencies for categorical parameters and mean, standard deviation (SD) and median for quantitative parameters. *p*-values ≤0.05 were considered as statistically significant. Statistical analysis was carried out using IBM SPSS Statistics 26® (IBM, Armonk, New York, USA).

## Results

### Patient characteristics

A total of 214 patients with ileostomy reversal were evaluated. Ninety-five patients who had ileostomy reversal without SPM® between January 2017 and December 2017 were compared to 119 patients who were operated with SPM® during the period of June 2018 to July 2020. There were 36 (37.9%) females in the group without SPM® and 50 (42.0%) females in the group with SPM® (*p* = 0.54). There was no difference in BMI, American Society of Anesthesiologists (ASA)-classification, anemia, diabetes, renal insufficiency, treatment with steroids or other immunosuppressive medication 6 weeks prior to operation between the two groups. More patients in the group with SPM® reported nicotine abuse: 25 (21.0%) vs. seven (7.4%) (*p* = 0.005). Overall, there were 44.4% patients in the trial who suffered from inflammatory bowel disease (Table 2).

**Table 2:** Baseline and surgical characteristics.

	Without SPM® (*n* = 95)	With SPM® (*n* = 119)	Total (*n* = 214)	*p*-value
Gender				0.54*
Female	36 (37.9%)	50 (42.0%)	86 (40.2%)	
Male	59 (62.1%)	69 (58.0%)	128 (59.8%)	
Age (years; mean ± SD)	50.9 ± 17.4	49.7 ± 19.0	50.2 ± 18.3	0.64**
BMI (kg/m^2^, mean ± SD)	23.5 ± 4.9	24.4 ± 4.7	24.0 ± 4.8	0.20**
ASA 1 and 2	74 (77.9%)	86 (72.3%)	160 (74.8%)	0.37*
Anemia	32 (33.7%)	46 (38.7%)	78 (36.4%)	0.45*
Nicotine abuse	7 (7.4%)	25 (21.0%)	32 (15.0%)	0.005*
Diabetes mellitus	7 (7.4%)	4 (3.4%)	11 (5.1%)	0.19*
Renal insufficiency	14 (14.7%)	25 (21.0%)	39 (18.2%)	0.22*
Inflammatory bowel disease	39 (41.1%)	56 (47.1%)	95 (44.4%)	0.34*
Treatment with steroids within 6 weeks prior to operation	2 (2.1%)	8 (6.7%)	10 (4.7%)	0.11*
Other immunosuppressive medication within 6 weeks prior to operation	6 (6.3%)	17 (14.3%)	23 (10.7%)	0.11*
Prior operation				0.37*
Low anterior resection	26 (27.4%)	30 (25.2%)	56 (26.2%)	
Proctocolectomy	18 (18.9%)	23 (19.3%)	41 (19.2%)	
Colectomy	11 (11.6%)	15 (12.6%)	26 (12.1%)	
Ileocecal resection	13 (13.7%)	17 (14.3%)	30 (14.0%)	
Right or left hemicolectomy	12 (12.6%)	13 (10.9%)	25 (11.7%)	
Small bowel resection	3 (3.2%)	10 (8.4%)	13 (6.1%)	
Sigmoid resection	8 (8.4%)	6 (5.1%)	14 (6.5%)	
Others	4 (4.2%)	5 (4.2%)	9 (4.2%)	
Intraoperative data				
Intraoperative serosa lesion	22 (22.1%)	19 (16.0%)	40 (18.7%)	0.25*
Intraoperative bowel perforation	5 (5.3%)	6 (5.0%)	11 (5.1%)	0.94*
Operating surgeon				
Head of department/Attending	32 (33.7%)	41 (34.5%)	73 (34.1%)	0.91*
Fellow	26 (27.4%)	35 (29.4%)	61 (28.5%)	
Resident	37 (38.9%)	43 (36.1%)	80 (37.4%)	
Duration of surgery (min; median ± SD)	68.0 ± 22.6	74.0 ± 30.5	70.0 ± 27.6	0.025***

Data are *n* (%) or mean ± SD. SD, standard deviation; SPM®, surgical procedure manager; BMI, body mass index; min, minutes.

* Chi-square test.

** *t*-test for independent samples.

*** Mann-Whitney-*U* test.

### Surgical characteristics

The operation performed most often prior to ileostomy reversal was low anterior rectal resection in 26.2%, followed by proctocolectomy in 19.2%. Regarding prior operations, there was no difference between the cohort without and with SPM® (*p* = 0.37). In terms of intraoperative complications, intraoperative serosal tear was detected in 22 (22.1%) patients without SPM® vs. 19 (16.0%) with SPM® (*p* = 0.25) and intraoperative bowel perforation in 5 (5.3%) vs. 6 (5.0%) (*p* = 0.94). Ileostomy reversal was performed by head of department/attendings in 34.1%, by fellows in 28.5% and by residents in 37.4% (*p* = 0.91). Operating time differed between patients without and with SPM®: 68.0 ± 22.6 min median vs. 74.0 ± 30.5 min median (*p* = 0.025) (Table 2).

### Postoperative outcome

Postoperative complications according to Clavien-Dindo (4) grade III–V occurred in 17 (17.9%) vs. 21 (17.6%) patients without vs. with the use of SPM® (*p* = 0.96). The rate of re-operations within 4 weeks after ileostomy reversal and surgical site infections did not differ between both groups (Table 3). Anastomotic leak occurred in nine (9.5%) patients without SPM® and in five (4.2%) patients with SPM® (*p* = 0.12). More patients without SPM® use developed an intraabdominal abscess: [ten (10.5%) patients vs. four (3.4%) patients; *p* = 0.035]. Postoperative bowel perforation (not involving the anastomosis) was seen in re-operation in five patients (5.3%) without SPM® vs. one (0.8%) patient with SPM® (*p* = 0.051). Intraoperative bowel perforation was not detected in one of these six patients.

**Table 3:** Postoperative complications without and with SPM®.

	Without SPM® (*n* = 95)	With SPM® (*n* = 119)	Total (*n* = 214)	*p*-value
Clavien-Dindo III–V	17 (17.9%)	21 (17.6%)	38 (17.8%)	0.96*
Reoperation within 4 weeks	14 (14.7%)	12 (10.1%)	26 (12.1%)	0.30*
Surgical site infection	12 (12.6%)	11 (9.2%)	23 (10.7%)	0.63*
Grade I	9 (9.5%)	6 (5.0%)	15 (7.0%)	
Grade II	1 (1.1%)	2 (1.7%)	3 (1.4%)	
Grade III	2 (2.1%)	3 (2.5%)	5 (2.3%)	
Ileus	10 (10.5%)	9 (7.6%)	19 (8.9%)	0.45*
Mechanical	7 (7.4%)	7 (5.9%)	14 (6.5%)	
Paralytic	3 (3.2%)	2 (1.7%)	5 (2.3%)	
Anastomotic leak	9 (9.5%)	5 (4.2%)	14 (6.5%)	0.12*
Intraabdominal abscess	10 (10.5%)	4 (3.4%)	14 (6.5%)	0.035*
Bowel perforation	5 (5.3%)	1 (0.8%)	6 (2.8%)	0.051*
Length of hospital stay (days; median ± SD)	6.0 ± 10.9	5.0 ± 5.3	5.0 ± 8.28	0.79**

Data are *n* (%) or mean ± SD. SD, standard deviation; SPM®, surgical procedure manager.

* Chi-square test.

** Mann-Whitney-*U* test.

**Table 4:** Multivariate analysis of postoperative complications without and with SPM®.

Independent variables	OR (95% CI)	*p*-value
Intraabdominal abscess (yes vs. no)	0.19 (0.05–0.71)	0.014*
Bowel perforation (yes vs. no)	0.09 (0.01–0.93)	0.043*

OR, odds ratio; CI, confidence interval.

* *p* ≤ 0.05.

Multivariate analysis showed a risk reduction for intraabdominal abscess {odds ratio (OR) 0.19 [95% confidence interval (CI) 0.05–0.71]; *p* = 0.014} and for bowel perforation [OR 0.09 (95% CI 0.01–0.93); *p* = 0.043] in the group with use of SPM® in ileostomy reversal. There was no difference in median length of hospital stay between the two cohorts: 6.0 ± 10.9 vs. 5.0 ± 5.3; *p* = 0.79 (Table 4).

## Discussion

To our knowledge, this is the first study to evaluate SPM® in visceral surgery. A comparison between no application of SPM® vs. application of SPM® in ileostomy reversal showed a lower rate of intraabdominal abscesses 10.5% vs. 3.4% and postoperative bowel perforations 5.3% vs. 0.8%. There was a difference in the operating time between patients without and with SPM®.

SPM® may reduce the risk for postoperative intraabdominal abscess and for bowel perforation in ileostomy reversal. SPM® has led to structured and standardized surgery. The steps and landmarks of ileostomy reversal were executed correctly. With the aid of SPM®, timing of antibiotic prophylaxis, glove change and subcutaneous wound irrigation before skin closure were administered. The substep of checking both the afferent and efferent small bowel loop for serosa or transmural lesions which can occur during adhesiolysis is a landmark step in SPM®. Reason for less postoperative intraabdominal abscesses could be that the attention even of experienced surgeons in this routine operation is increased by the optic and acoustic presentation of this landmark step by SPM®.

Standardization can lead to economization and improvement of intraoperative quality (5). In order to improve an existing process, new standards need to be set. Afterwards, the process can be consolidated through standardization and further be improved (6). A systematic review by Russ et al. showed that analogue standardized perioperative checklists reduced intraoperative mishaps and improved team communication in the operating room (7). In a retrospective cohort study with 1,189 patients with colorectal cancer, standardization of surgical procedures could reduce the rate of anastomotic leak, surgical site infection and reoperation. The standardization included laparoscopic approach combined with anastomosis for colon surgery and defunctioning ileostomy for low anterior rectal resection (8). The standardization of surgical equipment was effective in reducing costs in laparoscopic appendectomy (9).

The use of SPM® ensures that the operation is of a consistently high-quality standard and is performed in the same way by different surgeons (10). A substantial advantage is the segmentation of an operation in substeps. In our department, ileostomy reversal is divided in seven phases with 32 steps. SPM® navigates step-by-step through surgery by providing a checklist for each individual step during the procedure. The particular steps are provided both written and pictorial on the screen. This led to a more rapid training curve of surgical residents and scrub nurses. Everyone in the operating room knew the current and next step of the operation. This could improve the intraoperative course and reduced stress in the operating room. Some surgeons may fear the loss of their surgical individuality by using SPM®. This cannot be confirmed because a deviation from the steps can always be justified when necessary in the operative course.

Situational awareness can be compromised and the patient safety harmed if the surgeon is very focused and the communication between the different members of the operation team is missing (11). SPM® provides optic and acoustic presentation of the current and next step on the monitor. This can lead to enhanced situational awareness of all participating persons in the operating room (12). Thus, everyone can be aware of the current operating step, which is especially important in reducing distraction in difficult steps during the operation. The operating process is transparent for all persons in the operating room which is especially useful for the training of new operating room staff. This is important in times of staff shortage, fluctuation of the OR team which requires flexibility, and leasing of staff. There is a high process reliability with the help of SPM® because the intraoperative processes are repeatable and transparent.

In this study, duration of surgery was longer in the group with use of SPM®. This can be explained by the fact that at the time of implementation of SPM®, we also started to use intraoperative perfusion control of the anastomosis with indocyanine green (ICG). Hence, ICG perfusion check of the anastomosis has only been done in the SPM® group. It takes 5–10 min to set up the monitor and camera, to focus the anastomosis and to measure the perfusion. Measurement of ICG perfusion did not change intraoperative management in any patient because it did not lead to small bowel resection in any patient. Therefore, it is unlikely that it biased anastomotic leak rate. Each individual step of surgery is defined by an estimated time with the use of SPM®. This contributes to efficiency in the operating room because SPM® calculates the predicted end of surgery continuously which can support the team of anaesthesiologists in maintaining the time of anaesthesia. What is more, the transport and anaesthetic induction of the next patient can be timed efficiently. The difference between estimated and actual time is visualized.

So far, there is little literature about the benefits of SPM®. SPM® was first implemented in Germany in Leipzig in the early 2010s. Feige et al. evaluated the SPM® in functional endoscopic sinus surgery from March until October 2015. The average intervention time was shorter in the group with SPM® application. The variability of the operation was also decreased with SPM® (13). In primary total knee arthroplasty the mean surgical time could be reduced by around 10%–20% by using SPM® (14).

In this trial, SPM® contributed to patient safety in ileostomy reversal. The rate of severe postoperative complications (Clavien-Dindo III–V) between patients who underwent ileostomy reversal without and with SPM® was comparable. The overall rate of complications with 31.8% was comparable to the existing literature (15, 16). The rate of anastomotic leak was 9.5% in group without SPM® and 4.2% in the group with SPM®.

Several potential limitations of the trial must be taken into account. First, this was a retrospective trial. Data of the patients who were operated with SPM® were extracted retrospectively. After implementation of SPM® in 2018, ileostomy reversal was performed regularly with the help of this digital tool in our department. We chose the period from January 2017 to December 2017 for the comparison cohort because SPM® was not yet available at in our department. Second, we could not show a reduction in operating time with the use of SPM®. The use of intraoperative perfusion control of the anastomosis with indocyanine green in the SPM® group may has biased our results in this regard. We evaluated the operating time and postoperative outcome of ileostomy reversal in this trial from the start of implementation of SPM®. It takes a certain time until everybody is convenient with the new digital tool. This may have also contributed to the prolongation of operating time with SPM®. Third, there are always new interns who start their surgical career and who begin to perform ileostomy reversal at our department. We are convinced that the training curve of surgical residents can be steeper with the use of SPM®. Continuous feedback is important to improve the tool and to guarantee adherence to it. An integrated installation in the operation room will probably lead to better process optimization and better implication in the daily operating room routine.

Still, to our knowledge, this is the first study about this promising new technology enabling a digital standardization in visceral surgery.

## Conclusion

Surgical procedure manager (SPM®) provides a digital solution for standardization in the visceral surgery operating room. SPM® may reduce postoperative complications in ileostomy reversal such as intraabdominal abscess and bowel perforation. SPM® may contribute to patient safety.

## Data Availability

The raw data supporting the conclusions of this article will be made available by the authors, without undue reservation.
